# III-Nitride MEMS drum resonators on flexible metal substrates

**DOI:** 10.1038/s41378-025-00995-3

**Published:** 2025-10-24

**Authors:** A. Kassem, R. Gujrati, D. Bourrier, C. Ayela, F. Mathieu, I. Dufour, L. Nicu, V. Ottapilakkal, P. Vuong, S. Sundaram, W. Hunt, A. Ougazzaden, T. Leichlé, J. P. Salvestrini

**Affiliations:** 1 CNRS, IRL 2958 Georgia Tech-CNRS, Metz, France; 2 LAAS-CNRS, Université de Toulouse, CNRS, Toulouse, France; 3 Université de Bordeaux, Laboratoire IMS UMR-CNRS 5218, Talence, France; 4 Georgia Tech Europe, IRL 2958 Georgia Tech-CNRS, Metz, France; 5 Georgia Institute of Technology, School of Electrical and Computer Engineering, IRL 2958 Georgia Tech-CNRS, Atlanta, GA USA

**Keywords:** Electrical and electronic engineering, Materials science

## Abstract

We present a simple and efficient process for fabricating III-Nitride (III-N) mechanical resonators on flexible metal substrates. This method combines Van der Waals epitaxy of III-N epilayers with the deposition of a thick metal stressor atop the III-N layers. During thermal treatment, the 30 μm thick metal stressor deposited on a 300 nm AlGaN/500 nm GaN layer grown on a 3 nm two-dimensional hexagonal-Boron Nitride (2D h-BN) release layer, initiates a one-step Self-Lift-Off and Transfer (SLOT) process. This process effectively transfers the III-N heterostructure from the h-BN/Sapphire growth wafer to the flexible metal stressor substrate. Additional local etching of the metal stressor and deposition of front electrodes allow for releasing self-standing III-N layers with integrated actuation. Fabricated III-N MEMS drum resonators were analyzed using optical profilometry and laser Doppler vibrometer, enabling the observation of static deflections and distinct vibration modes. Finite element method (FEM) simulations were also performed to further understand experimental observations and assess the mechanical properties of the released III-N layers, particularly enabling the estimation of stress in the GaN and AlGaN released layers. This straightforward approach not only provides a practical solution for cost-effective III-N MEMS resonators but also ensures flexibility, and crack-free structures.

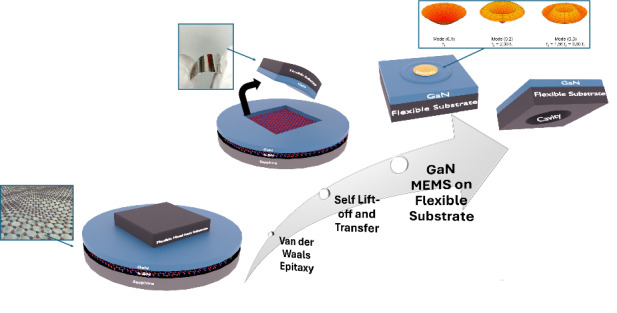

## Introduction

In recent years, the field of flexible electronics has undergone remarkable advancements, characterized by continuous improvements and innovations in manufacturing costs, weight reduction, and adaptability to various shapes at both material and device levels. This progress is driven by the anticipation of significant double-digit growth in the global market for flexible electronics, expected to reach a value of $74 billion by 2030^1^. Embracing the More than Moore paradigm, the integration of flexible electronics with sensors, actuators, and energy harvesters has resulted in enhanced device functionality, offering increased self-power provision, computing autonomy, and improved interaction with users and their environment. The current and envisioned applications range from wearable health monitoring devices for advanced healthcare and human–machine interfaces^2,3^ to the Internet of Things (IoT)^4^. A substantial array of sensors, actuators, and energy harvesters—including pressure sensors, motion sensors, micro-mirrors, temperature sensors, flexible piezoelectric generators, electromagnetic harvesters, and microfluidic pumps—are enabled by microelectromechanical systems (MEMS). These components are employed in implanted devices and are essential in diverse consumer electronics and automotive applications, where they are subjected to rigorous mechanical movements and harsh chemical environments^5–7^.

Gallium Nitride (GaN) is recognized for its unique properties, making it a promising material for a wide range of applications. Initially celebrated for its superior optoelectronic properties, leading to GaN-based blue diodes^8^, and outstanding radio frequency performance, including high-frequency integrated circuits exceeding 300 GHz^9^ and high-power applications over 10 W/mm^10^, GaN has also recently gained recognition for its excellent electromechanical properties^11^. Notably, GaN demonstrates significant piezoelectric^12^ and piezoresistive properties^13,14^, enabling its use in various transduction mechanisms. The formation of a two-dimensional electron gas (2DEG), at the interface of a thin AlGaN/GaN heterostructures, with high charge density (1 × 10^13^ cm⁻²) without intentional doping^15–18^, further distinguishes GaN from other piezoelectric materials. This unique feature broadens GaN’s applicability in reconfigurable systems and innovative sensors, thereby enhancing the versatility of GaN-based MEMS technology. Its high intrinsic wurtzite crystalline quality enhances mechanical performance^11^, while its wide bandgap of 3.4 eV^19^ makes it suitable for high-temperature operations^20,21^. GaN’s remarkable chemical inertness also provides resistance to corrosive environments^22^. These properties collectively position GaN as an ideal material for harsh environmental conditions, as well as for wearable and implanted sensors and actuators. Moreover, GaN’s potential for integrating MEMS resonators with high-gain electronics provides a significant advantage over other piezoelectric materials^10,23^.

Lately, various methods have been investigated to implement free-standing MEMS resonators onto flexible substrates. The release process from rigid growth substrate, in flexible MEMS fabrication can be achieved through etching of either the donor growth substrate or an intermediate sacrificial layer, each presenting trade-offs in process complexity, material compatibility, and device performance. While direct fabrication on flexible substrates eliminates transfer steps, it imposes significant limitations on material choice and film quality.

Direct fabrication on flexible substrates^24,25^, simplifies processing by eliminating additional transfer steps, reducing mechanical damage, and avoiding stress induced during release. It is particularly compatible with low-temperature deposition techniques. However, it suffers from major challenges, including surface roughness of polymer-based substrates like polyimide, which can degrade film uniformity and introduce defects. Additionally, material selection is constrained, as polycrystalline or amorphous films are typically used, leading to potential compromises in mechanical properties. Residual stress and warping are common issues due to the removal of the flexible substrate from a rigid carrier, while high-temperature growth processes, such as those required for III-Nitrides, are generally incompatible. Adhesion problems, delamination risks, and the deformation of flexible substrates during standard microfabrication steps further complicate the process, making it difficult to achieve high-performance MEMS devices.

Etching the donor substrate to render it flexible or enable layer transfer^26^, enables the fabrication of high-quality films, allowing for single-crystal or bulk-grown layers that enhance device performance. This method is well-suited for high-performance MEMS applications but presents several challenges. The etching process is complex, requiring precise control, and is often time-consuming and chemically intensive. Released ultra-thin layers can be fragile, making handling difficult and increasing the risk of mechanical failure. Furthermore, chemical residues from etching solutions can degrade transferred layers, affecting performance. Some commonly used substrates, such as sapphire (Al₂O₃), pose additional challenges due to their resistance to etching, necessitating specialized techniques. The standard etchant for sapphire is a highly corrosive mixture of sulfuric acid (H₂SO₄) and phosphoric acid (H₃PO₄), which requires elevated temperatures between 200 °C and 270 °C to achieve a controlled etching rate. However, the aggressive nature of this etchant, combined with the stringent process conditions, makes sapphire etching complex, hazardous, and often unsuitable for routine fabrication processes. The process is also costly, as donor substrates are sacrificed, and thinning-induced mechanical stress can lead to defects, reducing device reliability.

An alternative approach involves etching an intermediate sacrificial layer for layer transfer^27,28^, which allows for more controlled release and transfer of thin films. This technique reduces processing duration compared to full substrate etching, lowering fabrication costs. However, selecting an appropriate sacrificial layer is crucial to ensure compatibility with the active material. Non-uniform etching can result in surface roughness and stress concentration points, affecting device performance and bonding. Additionally, residual chemicals from the etching process may degrade the mechanical and electrical properties of the transferred layers. The original substrate may suffer damage during the release process, limiting its reusability, and some sacrificial layer removal methods require specific material properties, which restrict material choices. Further post-processing, such as planarization or bonding steps, may be necessary to improve surface uniformity and alignment, increasing fabrication complexity.

On the other hand, there has been a significant progress in fabricating high-performance free-standing MEMS resonators by transferring atomically thin two-dimensional semiconducting crystals (2D layer transfer-2DLT), such as hexagonal Boron Nitride (h-BN), graphene, molybdenum disulfide, and their heterostructures, onto pre-patterned substrates^29–32^. This methodology relies on the weak Van der Waals bonds between the 2D layer and its growth substrate to facilitate the lift-off and transfer of grown layers to an alternative host substrate. Alternatively, the Van der Waals epitaxy of III-N layers on 2D release layers can be used to lift-off and transfer thicker III-N layers. This approach has been successfully employed using h-BN as the 2D release layer on Sapphire to fabricate free-standing III-N MEMS on pre-patterned silicon substrates^33^, as well as flexible HEMTs gas sensors^34^, light-emitting diodes (LEDs)^35–37^, and solar cells^38^. Upon release of the device layer, this fabrication method allows for the recycling of the growth Sapphire wafer, thereby reducing both the environmental footprint and overall device cost.

Based on this approach, we present a simple and efficient wafer-scale transfer process for the production of III-N MEMS drum resonators onto flexible metal substrates. This process, termed “Self-Lift-Off and Transfer” (SLOT) consists in depositing a layer of metal stressor atop a III-N layer grown onto a 2D release layer and heating the substrate to provoke the delamination of the metal layer from the substrate, thus resulting in the III-N layer transfer to a flexible metal substrate. Additionally, the use of the metal stressor provides structural rigidity to prevent cracks during the lift-off process. Whereas the SLOT process was previously used for fabricating vertical InGaN LEDs and dense micro-LEDs arrays on large-area metal sheets^37,39^, here, freestanding MEMS drum resonators are fabricated by further etching the metal layer to release the mechanical structure. Top and bottom electrodes for MEMS actuation are provided respectively by the seed layer used to electrochemically grow the metal stressor layer and by a patterned metal layer deposited on the III-N MEMS active layer.

Several studies have explored the performance of drum resonators fabricated using various techniques and materials. For instance, nanoelectromechanical infrared detectors utilizing low-stress SiN deposition on silicon substrates exhibit excellent frequency stability with an Allan deviation of 5.5 × 10⁻⁷ and a responsivity of 343 W⁻¹^40^. Similarly, SiN-based drumhead optomechanical resonators fabricated via Low Pressure Chemical Vapor Deposition (LPCVD) and electron beam lithography achieve high-Q factors of 5.3 × 10⁶ at a frequency of 121.1 kHz^41^. Graphene-based drum resonators, fabricated using Chemical Vapor Deposition (CVD) and transferred onto pre-patterned silicon substrates, demonstrate MHz-range operation with moderate Q-factors (~416.6)^42^, while polymer-clamped graphene oscillators achieve frequency tunability of 14% at room temperature^43^. Additionally, AlN thin-film resonators, fabricated using RF magnetron sputtering, exhibit high-frequency operation within the 300 Hz–12 kHz range, with diaphragm deflections of 76 nm at ±20 V input^44^. Despite these advancements, the current literature lacks comprehensive data on yield, stress tolerance, and device uniformity so that a direct comparison of fabrication techniques, remains challenging due to differences in final substrates, material properties, and device architectures.

Using the SLOT process, we have successfully fabricated several hundred free-standing AlGaN/GaN (300 nm/500 nm thick bilayers) and GaN (340 nm thick layer) drum resonators over circular cavities created within 30 μm thick Cu and Ni metal substrates. Measurement of the static deflection of the III-N free-standing layers revealed a tensile and a compressive stress in the GaN and the AlGaN film, respectively. Next, we achieved integrated dynamic actuation of the GaN and AlGaN/GaN free-standing MEMS resonators and the eigenfrequencies and the shapes of the first resonant modes were observed by laser vibrometer. Finite element method (FEM) simulations using COMSOL were conducted to provide insights into the experimental observations and to estimate the stress within both layers. This technique offers a simple, efficient and cost-effective method for producing crack-free, free-standing III-N MEMS resonators, with significant potential for various applications, including wearable health monitoring, human-machine interfaces, and Internet of Things (IoT).

## Materials and methods

**Fig. 1:**
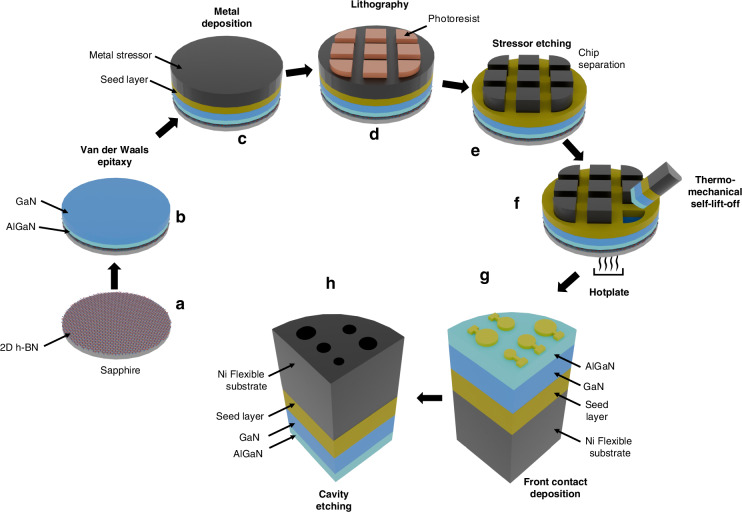
Different steps of the SLOT process for the fabrication of freestanding III-N films on flexible metal substrates. **a** MOVPE growth of a 3 nm 2D h-BN release layer on a 2-inch Sapphire growth substrate. **b** Van der Waals epitaxy of III-N epilayer. **c** Deposition of metal seed layer followed by electrodeposition of a 30 μm-thick stressor layer of Ni or Cu (Nickel or Copper). **d**, **e** 1 cm^2^ chips definition and separation by photolithography and wet etching of the metal layer. **f** Thermally activated mechanical self-exfoliation (SLOT) of the chips. **g** Deposition and patterning of the metal front contact on the III-N layer, followed by **h** the release of the III-N membrane on the opposite side through etching of the metal

The schematic representation of the SLOT process utilized for fabricating self-standing III-N MEMS drum resonators on flexible metal substrates is illustrated in Fig. 1.

Epitaxial structures were synthesized on 2-inch c-plane (0001)-oriented epi-ready sapphire (Al₂O₃) substrates, commercially procured, using a metal-organic vapor phase epitaxy (MOVPE) system equipped with a Close Coupled Showerhead (CCS) reactor configuration (Aixtron CCS 3×2″). Prior to growth, substrates underwent a standard solvent cleaning sequence involving acetone, isopropanol, and deionized water, followed by nitrogen blow-drying to eliminate residual moisture. Throughout the entire growth process, high-purity hydrogen (H₂) was utilized as the carrier gas. As chemical precursors, triethylboron (TEB), trimethylgallium (TMGa), and trimethylaluminum (TMAl) were employed to supply boron, gallium, and aluminum, respectively. Ammonia (NH₃) served as the nitrogen source. The growth commenced with the deposition of a 3 nm-thick two-dimensional hexagonal boron nitride (h-BN) interlayer at a substrate temperature of 1280 °C (Fig. 1a). The deposition occurred in a hydrogen ambient maintained at 90 mbar, with a TEB flow rate of 60 μmol/min, resulting in a growth rate of approximately 15 nm/h. Following the h-BN nucleation layer, a 300 nm-thick Si-doped Al₀.₁₄Ga₀.₈₆N buffer layer (Al mole fraction of 14%) was grown at 1100 °C. The aluminum content was precisely controlled via calibrated TMAl and TMGa flow rates, and silane (SiH₄) was introduced as the n-type dopant source. Subsequently, a 500 nm-thick Si-doped GaN top layer was deposited on the AlGaN buffer under identical thermal conditions, targeting a doping concentration of 5 × 10¹⁸ cm⁻³^34,45^ (Figs. 1b and S1a).

Post-growth, the samples were extracted from the reactor for metallization steps. The surface of the n-type GaN layer was prepared by sequential deposition of dual-layer metal stacks—Ti (25 nm)/Au (25 nm) and Ti (100 nm)/Au (200 nm)—using a combination of electron-beam evaporation and sputtering techniques. These layers served as seed and adhesion layers for subsequent electroplating of a 30 μm-thick metal stressor, either copper (Cu) or nickel (Ni) (Figs. 1c and S1b). Chip patterning was achieved via ultraviolet (UV) photolithography using a positive-tone photoresist (SPR 220-3). After an organic clean and dehydration bake at 115 °C for 10 min, the photoresist was spin-coated at 4000 rpm for 40 s, soft-baked at 115 °C for 90 s, and UV-exposed at 365 nm with a dose of 150 mJ/cm^2^. A post-exposure bake (PEB) at 115 °C for 90 s preceded development in MF-319 developer for 30 s. This process defined nine individual 12 × 12 mm² chips across the wafer surface (Fig. 1d). This photoresist acts as a protective layer during the local removal of the metal layer by wet etching, resulting in the separation of the nine chips. Selective etching of the patterned metal stressor layer was carried out using isotropic wet chemical etching. Both Cu and Ni were locally removed using a solution comprising 50 ml of HCl, 28 ml of H₂O₂, and 22 ml of deionized water. Residual photoresist was subsequently stripped using a dimethyl sulfoxide (DMSO) remover at 80 °C (Figs. 1e and S1c).

The Self-Lift-Off process of the III-Nitride epitaxial layers was thermally initiated by placing the entire wafer structure on a hotplate at 100 °C in ambient air for 1 h. Due to the mismatch in thermal expansion coefficients between the metal stressor (Cu^46^ : 16.5 × 10⁻⁶ K⁻¹; Ni^47^ : 13.4 × 10⁻⁶ K⁻¹) and the Sapphire substrate^48^ (8.1 × 10⁻⁶ K⁻¹), significant biaxial thermal stresses were generated. This mismatch resulted in compressive stress accumulation in the metal layer and tensile stress within the Sapphire. At the periphery of each chip, edge effects gave rise to localized shear and normal stresses at the metal/Sapphire interface. These stresses, coupled with the inherently weak Van der Waals bonding of the 2D h-BN interlayer, led to delamination once a critical interfacial shear stress was reached—experimentally observed to initiate at approximately 89 °C^39^. Crack propagation advanced from the edges inward, ultimately leading to complete lift-off of the AlGaN/GaN epitaxial structure, now supported on the flexible electroplated metal substrate (Figs. 1f and S1d).

For some chips, the AlGaN buffer was selectively removed via dry etching using an inductively coupled plasma (ICP) etching system. A gas mixture of Cl_2_, Ar and BCl was employed. An 80-s etch duration resulted in a final GaN layer thickness of approximately 340 ± 30 nm, as confirmed by profilometric analysis. Subsequent electrode patterning on the exposed GaN or AlGaN surfaces began with organic cleaning and dehydration, followed by photolithography using a negative-tone photoresist (AZ nLOF 2020) spin-coated at 4500 rpm for 40 s. The coated samples were soft-baked at 110 °C for 1 min, exposed to UV light at a dose of 65 mJ/cm², then subjected to a PEB at 110 °C for 1 min before developing in MF-319 for 60 s. This was followed by an oxygen plasma cleaning step and buffered oxide etch (BOE) treatment to remove oxides. Metal contacts comprising Ti (30 nm)/Au (100 nm) were deposited by electron-beam evaporation, and lift-off was performed using DMSO at 80 °C (Fig. 1g). To finalize the device structure, circular cavities were defined in the metal stressor layer by photolithography (SPR 220-3, 4000 rpm, 40 s), with a soft bake at 115 °C for 90 s, UV exposure at 150 mJ/cm², development in MF-319 for 30 s, and a final hard bake at 100 °C for 10 min. These cavities were subsequently etched using the same diluted HCl/H₂O₂ solution as before. Throughout the process, the Ti/Au seed layer was preserved to function as the bottom electrode (Fig. 1h). The resulting freestanding circular devices—comprising either 300 nm AlGaN/500 nm GaN or 340 ± 30 nm GaN films—featured integrated top and bottom electrodes, rendering them fully functional for subsequent actuation experiments (Fig. S2).

**Fig. 2:**
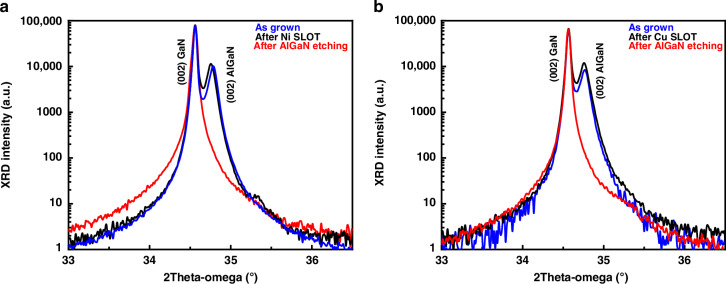
Structural characterization of III-N layers before and after transfer. High-resolution X-ray diffraction (HR-XRD) 2θ–ω scans of MOVPE-grown III-N heterostructures are shown for: **a** 300 nm AlGaN/500 nm GaN layers on 3 nm h-BN/Sapphire before transfer (blue curve); after SLOT transfer onto Nickel (black curve); and after AlGaN removal, leaving a ~340 nm GaN layer (red curve). **b** Corresponding scans for the same sequence on Copper substrate. The scans demonstrate retention of crystalline quality and the thinning of the structure post-etching

Figure 2 shows the high-resolution X-ray diffraction (HR-XRD) 2θ-ω scans of the AlGaN/GaN heterostructure after MOVPE growth (before metal deposition), after SLOT process (on exposed AlGaN after chip lift-off), and after AlGaN dry etching (for GaN MEMS), for both Nickel (Fig. 2a) and Copper (Fig. 2b) substrates. Diffraction peaks observed at 34.5° and 34.7° correspond to the (002) reflections of GaN and AlGaN layers, respectively. No or very little shift of the peaks is observed after the SLOT process for both Nickel and Copper substrates. This is expected since the absence of dangling bonds on the 2D h-BN surface enables Van der Waals epitaxy (VWE) of III-N layers, resulting in reduced residual stress in the epilayer due to its weak bonding with the 2D layer, thereby minimizing stress in the AlGaN buffer layer. This observation indicates that Van der Waals epitaxy results in nearly stress-free III-N epilayers and shows the preservation of crystallographic properties during the SLOT process. The small shift of the AlGaN peak in the HR-XRD 2θ-ω scans observed after the SLOT process can be attributed to the inhomogeneity in Aluminum content across the epilayers^49^, since the XRD scans were conducted at different locations on the sample before and after SLOT process. The HR-XRD 2θ-ω scan for the GaN layers shows the complete disappearance of the AlGaN peak, confirming the complete etching of this layer.

**Fig. 3:**
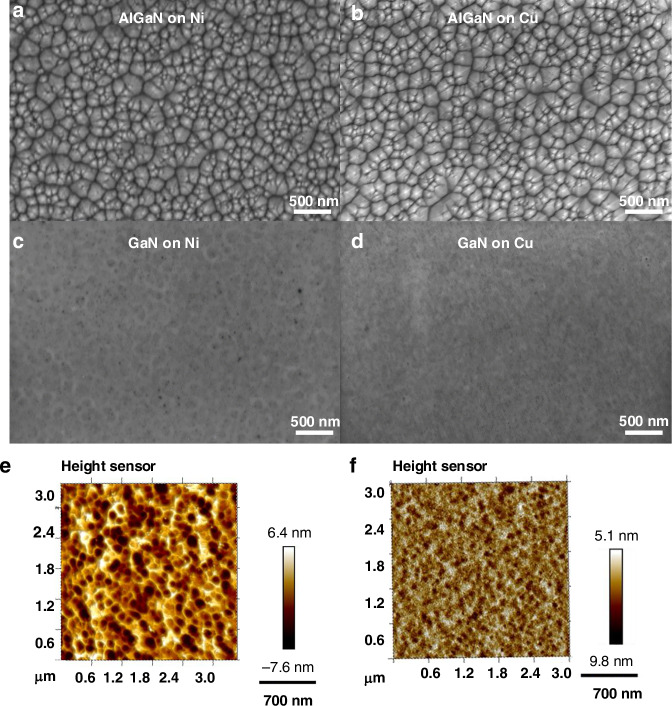
Surface morphology of III-N films after SLOT transfer and AlGaN etching. **a** SEM image of the AlGaN surface after SLOT transfer onto Nickel. **b** SEM image of AlGaN surface on Copper. **c** SEM image of GaN surface after AlGaN etching on Nickel. **d** SEM image of GaN surface after AlGaN etching on Copper. **e** AFM image (3 × 3 μm²) of GaN on Nickel showing ~2 nm RMS roughness. **f** AFM image (3 × 3 μm²) of GaN on Copper showing ~1.8 nm RMS roughness. All surfaces demonstrate nanoscale smoothness suitable for MEMS applications

Scanning electron microscopy (SEM) observations of the AlGaN layer surface after transfer on the metal substrate reveal a granular morphology on both Nickel (Fig. 3a) and Copper (Fig. 3b) substrates, which corresponds to the expected AlGaN morphology grown on 2D h-BN layer^34^. Additionally, SEM observations of the GaN surface reveal the morphology following ICP etching (Fig. 3c, d). Figure 3e, f showcases the surface morphology of a 340 nm GaN layer post-AlGaN dry etching. The root mean square (RMS) value of surface roughness is approximately 2 nm for GaN on Nickel substrate (Fig. 3e) and 1.8 nm for GaN on Copper substrate (Fig. 3f).

**Fig. 4:**
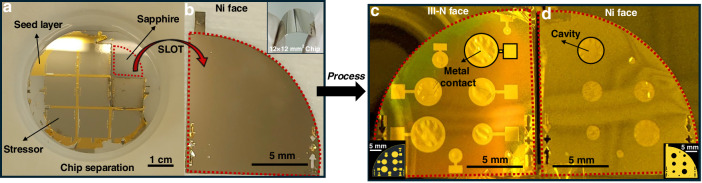
Visual demonstration of the SLOT process and final device configuration. **a** Photograph of eight 12 × 12 mm² III-N chips grown on a 2-inch sapphire wafer. **b** Photograph of a single chip after SLOT release (inset: a flexible 1 cm² III-N membrane on Ni, showing mechanical compliance). **c** View of the III-N side after cavity etching, showing the geometry and placement of top metal electrodes. **d** View from the Nickel side, highlighting the etched stressor cavities that define the suspended membrane areas

In both cases, the use of a 30 μm-thick stressor metal layer effectively reinforces the structure during Self-Lift-Off process (while simultaneously maintaining flexibility (inset Fig. 4b), thereby ensuring the consistent fabrication of crack-free flexible self-standing III-N MEMS, as it can be seen in Fig. 4 for the devices fabricated on Ni substrates. Figure 4a shows the Sapphire wafer after Nickel etching for chip separation and Fig. 4b displays a single chip after its release using the SLOT process. Fabricated III-N MEMS, after front electrode deposition and etching cavities into the metal substrate, are shown in Fig. 4c, d. Circular MEMS of various diameters were obtained by etching various size cavities (diameters of 100, 200, 500 μm and 1, 1.5, and 2 mm) and the top electrodes were designed with a diameter larger than the cavity to cover entirely the released III-N membranes. One should note that cavities larger than 500 μm remained highly fragile and susceptible to damage, consequently only the III-N MEMS with a diameter <500 μm were fully characterized. Images of III-N MEMS on Cu substrates are provided in the supplementary information in Fig. S3b, c. Because of the high built-in stress within the electroplated Copper layer, the Copper substrates show a tendency to curl upon lift-off which impedes the characterization of the fabricated III-N MEMS drum resonators on Copper. This underlines the critical role of the metal substrate for the fabrication of III-N MEMS using the presented process. While the SLOT process was initially developed using a Copper stressor for fabricating vertical InGaN light-emitting diodes (LEDs) and dense micro-LED arrays on large-area free-standing membranes^37,39^, low stress Nickel substrate is better suited for the fabrication of mechanical structures.

## Results

**Fig. 5:**
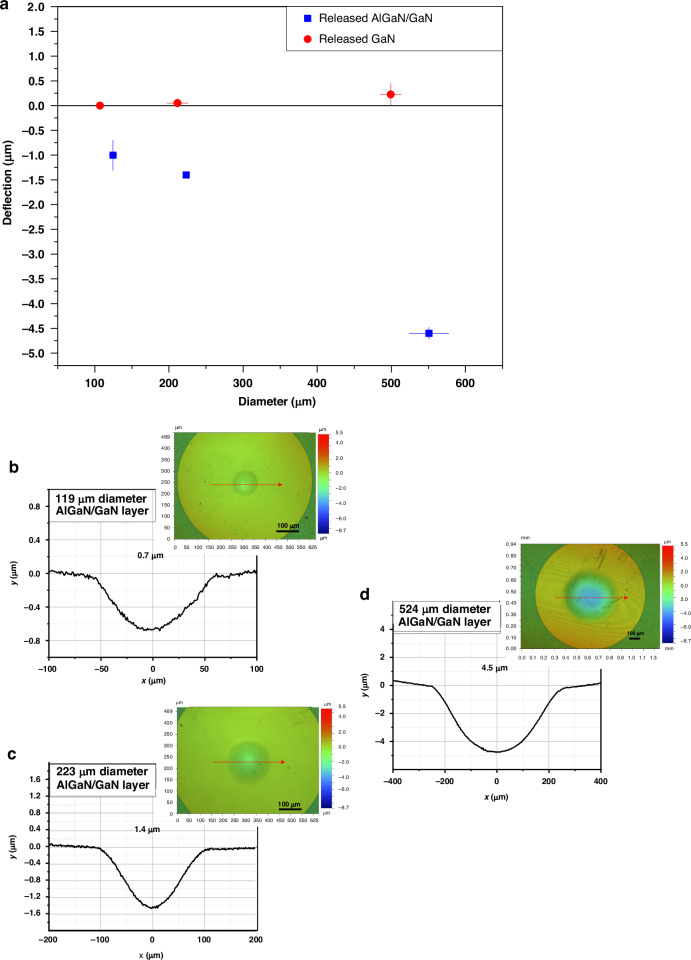
Static deflection profiles of released III-N membranes as a function of membrane size. **a** Plot of maximum static deflection at membrane center versus membrane diameter. Measurements from two devices per size are shown; horizontal error bars indicate variation in measured diameters. **b** Profilometry of a 119 μm diameter AlGaN/GaN membrane showing ~0.7 μm deflection. **c** Profilometry of a 223 μm membrane with ~1.4 μm deflection. **d** Profilometry of a 524 μm membrane showing ~4.5 μm deflection. Insets show optical profilometry maps with electrode outlines for spatial reference

Static and dynamic characterizations of the III-N MEMS drum resonators on Nickel substrate were conducted. Static profiling results obtained by optical profilometry (Veeco NT9080) are shown in Fig. 5. Figure 5a presents the maximum deflection of released GaN and AlGaN/GaN layers as a function of cavity diameter. While in both cases the deflection increases with increasing cavity diameter, GaN membranes exhibit small deflections compared to AlGaN/ GaN membranes: deflections of 0.7 μm for a 100 μm diameter cavity (Fig. 5b), 1.4 μm for 200 μm (Fig. 5c), and 4.5 μm for 500 μm (Fig. 5d) are measured. The relative flat profiles of the released GaN films suggest that GaN layers are either free of stress or experience tensile stress. Upon the presence of the additional AlGaN layer, larger deflections indicate that the AlGaN layer is compensating for the tensile stress within the GaN layer. This compensation suggests that the AlGaN layer is under compressive stress. It is worth noting that the diameters of the cavities derived from the optical profile are 10–20% larger than expected (Fig. 5, confirmed by optical and SEM observations, as shown in Fig. S4). This discrepancy is attributed to the isotropic nature of the metal manual wet etch process (in 50 ml HCl, 28 ml H₂O₂ and 22 ml H_2_O) and the considerable thickness of the metal stressor (30 μm) that needs to be etched. Additionally, some cavities exhibit an oval shape rather than a circular one, which is attributed to the flexible nature of the substrate that can result in deviations in pattern transfer from the original UV mask pattern during photolithography.

**Fig. 6:**
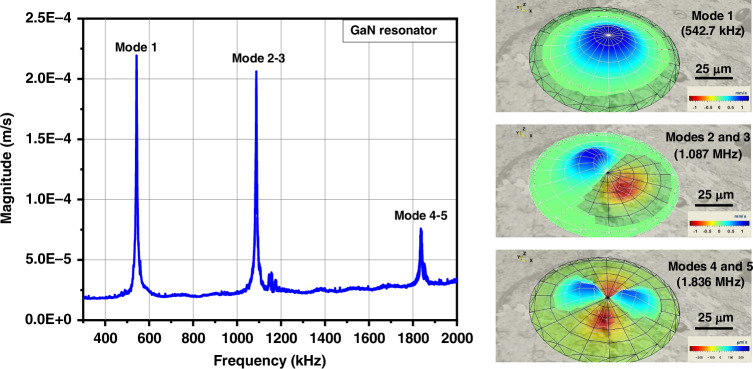
Typical resonance spectrum of a 340 nm thick, 107 µm diameter GaN MEMS and its corresponding vibration modes. Amplitude of vibration is measured at the location of the largest displacement

**Fig. 7:**
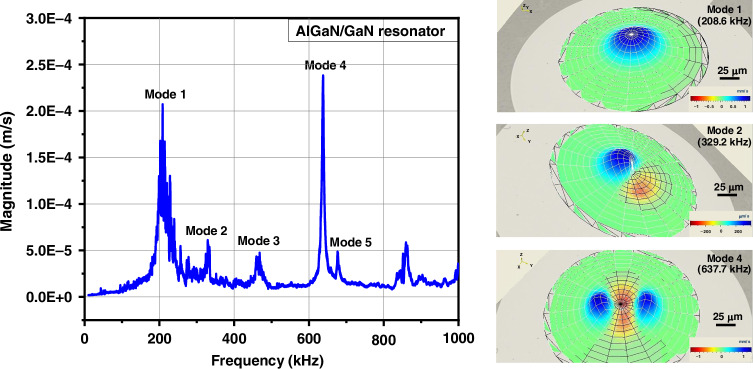
Typical resonance spectrum of a 300 nm/500 nm thick and 223 µm in Nickel cavity diameter AlGaN/GaN resonator and its corresponding vibration modes. Amplitude of vibration is measured at the location of the largest displacement

The dynamic characterization of the GaN and AlGaN/GaN resonators was carried out using integrated actuation and laser Doppler vibrometer (MSA-500 Micro System Analyzer from Polytec). In this endeavor, the MEMS drum resonators were stimulated by applying an AC voltage of ∼70 mV to the III-N membrane using the set of electrodes (top electrode patterned onto the III-N film and bottom electrode consisting of the seed layer), and the velocity of MEMS membrane was mapped while sweeping the AC frequency. Figure 6 presents the dynamic response of a 340 nm thick and 107 µm in diameter GaN resonator, and Fig. 7 shows the response of a 300 nm/500 nm thick and 223 µm diameter AlGaN/GaN resonator. Resonant frequency peaks for the first five modes can clearly be identified. As expected, the first mode exhibits a symmetric deflection pattern with a broad central displacement. For the GaN resonator, the second and third modes are ideally degenerate, whereas for the AlGaN/GaN resonator non-degeneration and frequency splitting are observed, which can be attributed to the geometrical deviation from a circular membrane (oval cavity). Similarly, the fourth and fifth modes also exhibit non-degeneracy due to imperfections of the AlGaN/GaN resonator geometry. These non-degenerated modes were also observed on a 209 µm diameter GaN resonator that displays a slightly oval shape (Fig. S5).

The MEMS fundamental resonance mode was also excited when driving a DC voltage with an AC voltage at half the resonance frequency, as can be seen from the resonance peak observed in Fig. S6a, b. This result tends to indicate an electrothermal actuation mechanism since the thermal power dissipation includes a dynamic component at 2ω^50^ (Eq. (1) in the Supplementary Information). AlGaN and GaN have a wurtzite crystalline structure and are thus piezoelectric materials; hence, we cannot completely rule out a possible contribution of the inverse piezoelectric effect to the overall structure actuation. However, this effect is likely to be small because the III-N layers are doped and display low resistivity, and because the presence of the electrode on the entire surface of the membrane is not likely to induce bending moments that are required to deform the membrane and excite flexural vibrations. (More details about the actuation mechanisms are provided in the Supplementary Information in Section 3, “Actuation mechanisms of the fabricated resonators”).

To substantiate the hypothesis of a tensile stress in the GaN and a compressive stress in the AlGaN layer, deduced from optical profilometry, we conducted a comparative analysis of the experimental resonance frequencies of the resonators with the eigenfrequencies calculated by finite element method (FEM) simulations. FEM simulations of self-standing GaN and AlGaN/GaN circular structures, including the top and bottom metal electrodes, were conducted using COMSOL. Values of the Young’s modulus, the Poisson’s ratio and mass density for AlGaN and GaN were obtained from the literature (*E*_AlGaN_ = 261 GPa^53^, *E*_GaN_ = 285 GPa^51^, *ν*_AlGaN_ = 0.2^52^, *ν*_GaN_ = 0.18^53^, *ρ*_AlGaN_ = 5304 kg/m³, and *ρ*_GaN_ = 6150 kg/m³). The simulations were performed using clamped boundary conditions, with internal stresses used as an adjusting parameter for the GaN and AlGaN resonators. Under no-stress conditions, the experimental resonant frequencies for GaN resonators were found to be much higher than the simulated ones, indicating that the GaN layer is under tensile stress. This is confirmed by the absence of static deformation in the GaN released film, as observed under the optical profilometer (Fig. 5a). In contrast, for the AlGaN/GaN heterostructure, the experimental resonant frequencies were relatively close to the simulated ones under no-stress conditions. This suggests that the tensile stress in the GaN layer is nearly compensated by the compressive stress in the AlGaN layer. When incorporating the internal stress as an adjustable parameter in our FEM simulations, the computed resonance frequency of various size membranes was found to be in agreement with experimental values for an average tensile stress of approximately 130 MPa in the GaN layer and a compressive stress of around −120 MPa in the AlGaN layer (see Table S1 in the Supporting Information).

To confirm this analysis, we have assessed the influence of the internal stress on the resonance through the theoretical expression for the resonant frequency of a monolayer circular structure. In the general case of plates and membranes, this resonant frequency can be described as follows^54–56^:

1 $${f}_{{ij}}=\frac{{\lambda }_{{ij}}^{2}}{2\pi }\frac{h}{{R}^{2}}\sqrt{\frac{E}{12\left(1-{\nu }^{2}\right)\rho }}\sqrt{1+\frac{12\left(1-{\nu }^{2}\right){\beta }_{{ij}}^{2}\sigma {R}^{2}}{{\lambda }_{{ij}}^{4}E{h}^{2}}}$$

Where *R* and *h* are the structure radius and thickness, *E* is the Young’s modulus, *ν* is the Poisson’s ratio, *ρ* is the volumetric mass density, *σ* is the internal stress and *λ*_*ij*_ and *β*_*ij*_ are constants that depend on the mode number (with *i*, the number of nodal circles including the plate boundary and *j*, the number of nodal diameters)^57^. When the internal stress is negligible compared to the elastic effect, such structures behave as plates and the resonance frequency is inversely proportional to the square of the radius ${R}^{2}$:

2 $${f}_{{ij}}=\frac{{\lambda }_{{ij}}^{2}}{2\pi }\frac{h}{{R}^{2}}\sqrt{\frac{E}{12\left(1-{\nu }^{2}\right)\rho }}$$

When the internal stress dominates the elastic effect, structures behave as membranes and the resonance frequency is inversely proportional to the radius $R$:

3 $${f}_{{ij}}=\frac{{\beta }_{{ij}}}{2\pi R}\sqrt{\frac{\sigma }{\rho }}$$

Transition between the plate to membrane behavior occurs for:

4 $${\sigma }_{{{\rm{transition}}}}=\frac{{\lambda }_{{ij}}^{4}}{{\beta }_{{ij}}^{2}}\frac{E{h}^{2}}{12\left(1-{\nu }^{2}\right){R}^{2}}$$

So pure plate and membrane behaviors are expected when $\sigma < \frac{{\sigma }_{{{\rm{transition}}}}}{10}$ and $\sigma > 10\,{\sigma }_{{{\rm{transition}}}}$, respectively.

**Fig. 8:**
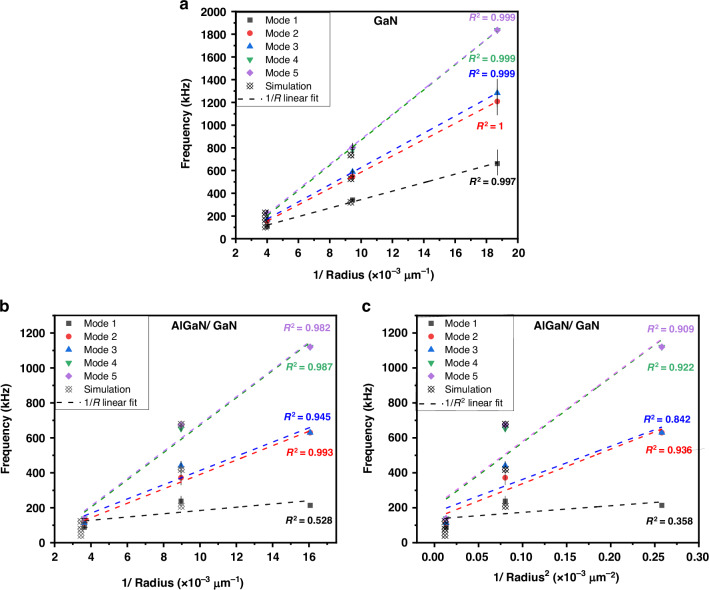
Experimental resonant frequencies of the first five modes and simulated values obtained by FEM. **a** For released GaN on 100, 200, and 500 μm diameter Ni cavities, plotted as a function of the inverse of the structure radius; **b** for released AlGaN/GaN heterostructures on 100, 200, and 500 μm diameter Ni cavities, plotted as a function of the inverse of the structure radius; and **c** for released AlGaN/GaN heterostructures on 100, 200, and 500 μm diameter Ni cavities, plotted as a function of the square of the inverse of the structure radius. Theoretical FEM resonance frequencies are calculated for GaN and AlGaN/GaN, considering compressive stress in AlGaN and tensile stress in GaN

The experimental and simulated resonance frequencies of the GaN MEMS are plotted in Fig. 8a as a function of the inverse of the radius 1/*R*. A clear linear dependency of the resonance frequencies is observed, thus indicating a membrane-like behavior. The values of the computed transition stress for the various diameters of GaN MEMS using modified Eqs.(1)–(4) to take into account the metal/GaN/metal multilayer (*σ*_transition_ = 18 MPa and *σ*_transition_ = 3 MPa for the fundamental mode of a 211 µm and a 499 µm diameter structure, respectively), were found to be at least a magnitude lower than the stress estimated by simulation (Table S1), thus confirming the observed membrane-like behavior.

Analogously, we have plotted the experimental and simulated resonance frequencies of the AlGaN/GaN MEMS as a function of the inverse of the radius, 1/*R*, and as a function of 1/*R*^2^ (Fig. 8b, c). However, in this case, there is no clear dependency and the AlGaN/GaN structure cannot be considered as a pure plate or membrane. By modifying Eqs. (1)–(4) to take into account the AlGaN/GaN bilayer, and using the internal stress value for the GaN layer estimated by FEM (130 MPa), the theoretical transition stress is calculated to be −114 MPa and −200 MPa for the fundamental mode of a 223 µm and a 550 µm diameter structure, which is in the same order of magnitude of the stress estimated by simulation (−120 MPa), thus confirming the experimental observations that the AlGaN/GaN MEMS does not exhibit a pure plate-like or membrane-like behavior, as well as the values for estimated internal stress.

## Conclusions

In conclusion, this study demonstrates the simplicity of the SLOT process for fabricating flexible III-N MEMS resonators, utilizing a h-BN release layer grown simultaneously with the III-N active MEMS epilayers. By eliminating the need for external force to transfer III-N layers from the sapphire growth substrate, this approach represents a valuable addition to the arsenal of 2D material-based mechanical lift-off and transfer (2DLT) techniques.

Several hundred of resonators have been successfully fabricated using the SLOT process, yielding promising results in terms of quality factors, with values of *Q* = 153 (Mode 1), *Q* = 385 (Modes 2–3), and *Q* = 313 (Modes 4–5) for the 107 μm GaN resonator, and *Q* = 80 (Mode 1), *Q* = 56 (Mode 2), *Q* = 42 (Mode 3), *Q* = 178 (Mode 4), and *Q* = 264 (Mode 5) for the 223 μm AlGaN/GaN resonator (see Fig. S7 in SI). Additionally, the integrated dynamic actuation of GaN and AlGaN/GaN III-N MEMS drum resonators fabricated by the SLOT process has been demonstrated, showing clean resonant modes consistent with simulations. Moreover, the tensile stress of the GaN film and the compressive stress of the AlGaN film were also estimated.

However, slight process variations were observed. These variations can be attributed to several factors, including the isotropic nature of the wet manual etching step used for stressor patterning, the embedding of III-N layers, and the flexibility of the entire chip. In the next generation of MEMS devices, we anticipate addressing these issues through several refinements to the process. These improvements will pave the way for more reliable and robust devices for dynamic applications. Furthermore, future studies will be crucial for further evaluating the bendability and torsional tolerance of these resonators to assess their robustness in dynamic environments.

This transfer method not only facilitates the creation of lightweight, flexible, and crack-free structures but also offers potential for flexible GaN MEMS resonators on various metal substrates.

## Supplementary information

Supplemental Material Supplementary Information

## Data Availability

The data that support the findings of this study are available from the corresponding author upon reasonable request.
