# Supplementary material for: III-Nitride MEMS drum resonators on flexible metal substrates
Source: Microsyst Nanoeng. 2025 Oct 24;11:197. doi: 10.1038/s41378-025-00995-3 (PMC12552455; doi:10.1038/s41378-025-00995-3)
Supplement: Supplementary file 1 — Supplemental Material Supplementary Information [file 41378_2025_995_MOESM1_ESM.docx]

Supporting Information

III-Nitride MEMS Drum Resonators on Flexible Metal Substrates

A. Kassem^1^, R. Gujrati^1^, D. Bourrier^2^, C. Ayela^3^, F. Mathieu^2^, I. Dufour^3^, L. Nicu^2^, V. Ottapilakkal^1^,

P. Vuong^1,4^, S. Sundaram^1,4^, W. Hunt^5^, A. Ougazzaden^1,5^, T. Leichlé^1,2,5*^, J.P. Salvestrini^1,4,5*^

^1^CNRS, IRL 2958 Georgia Tech-CNRS, Metz, France

^2^LAAS-CNRS, Toulouse, France

^3^Université de Bordeaux, Laboratoire IMS UMR-CNRS 5218, Talence, France

^4^Georgia Tech Europe, IRL 2958 Georgia Tech-CNRS, Metz, France

^5^Georgia Institute of Technology, School of Electrical and Computer Engineering, IRL 2958 Georgia Tech- CNRS, Atlanta, GA, USA

* Corresponding Authors: Jean-Paul.Salvestrini@georgiatech-metz.fr; [thierry.leichle@cnrs.fr](mailto:thierry.leichle@cnrs.fr)

# **Section 1:** SLOT process main steps

(b)

(d)


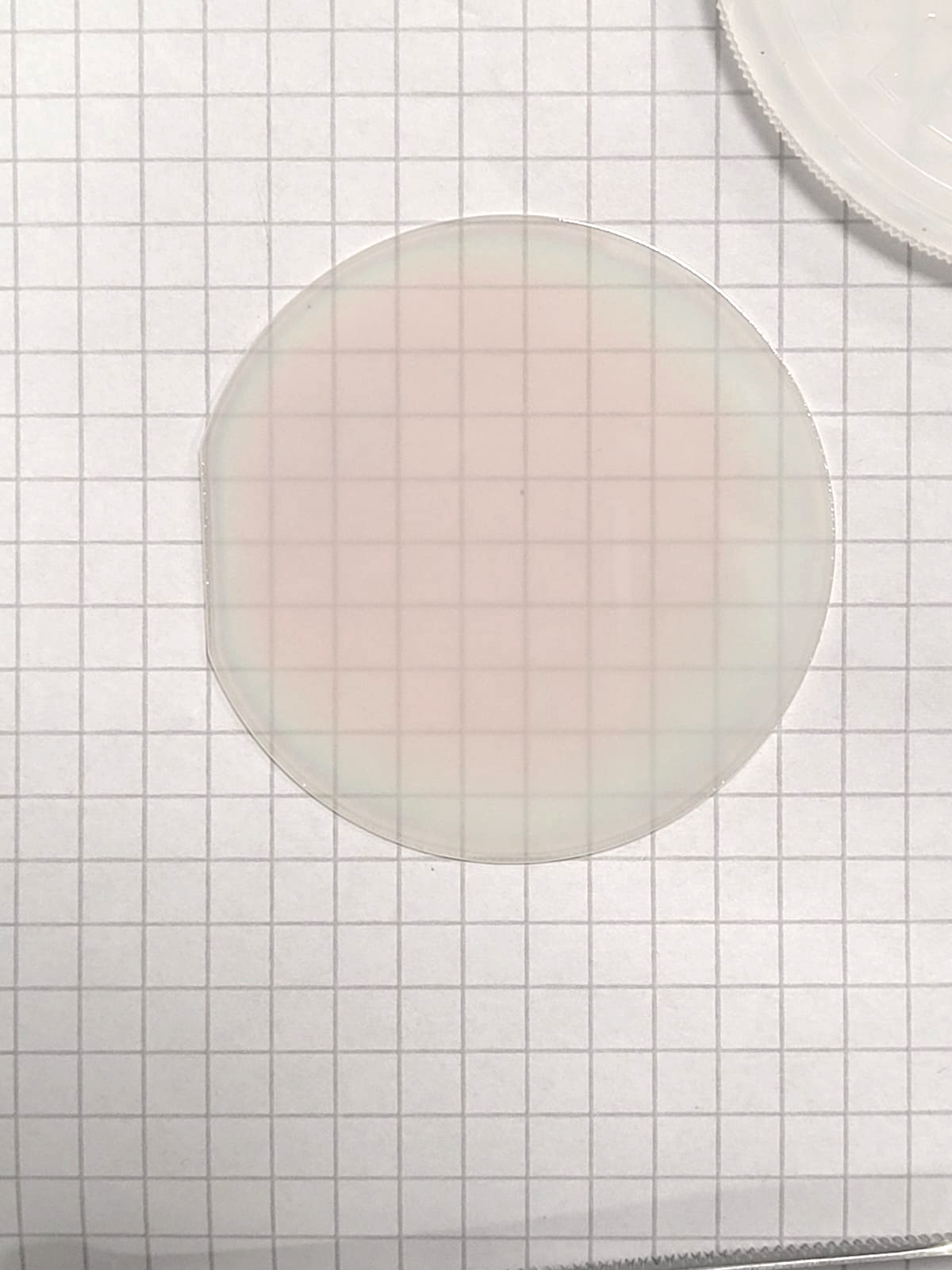

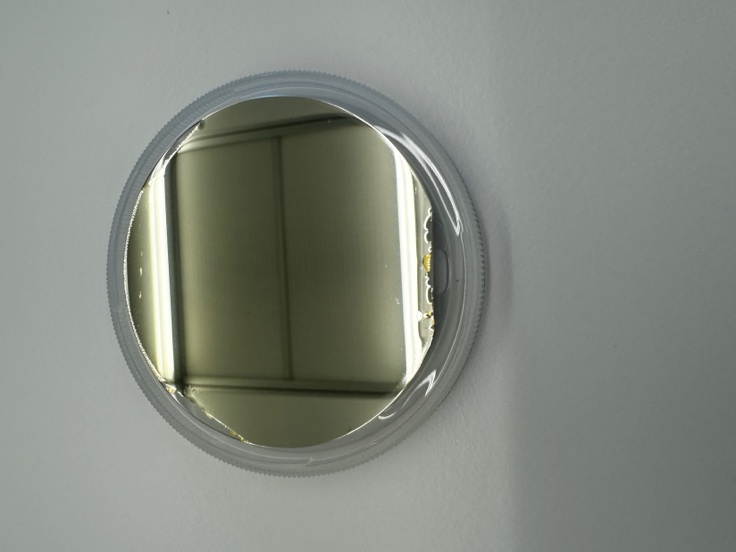

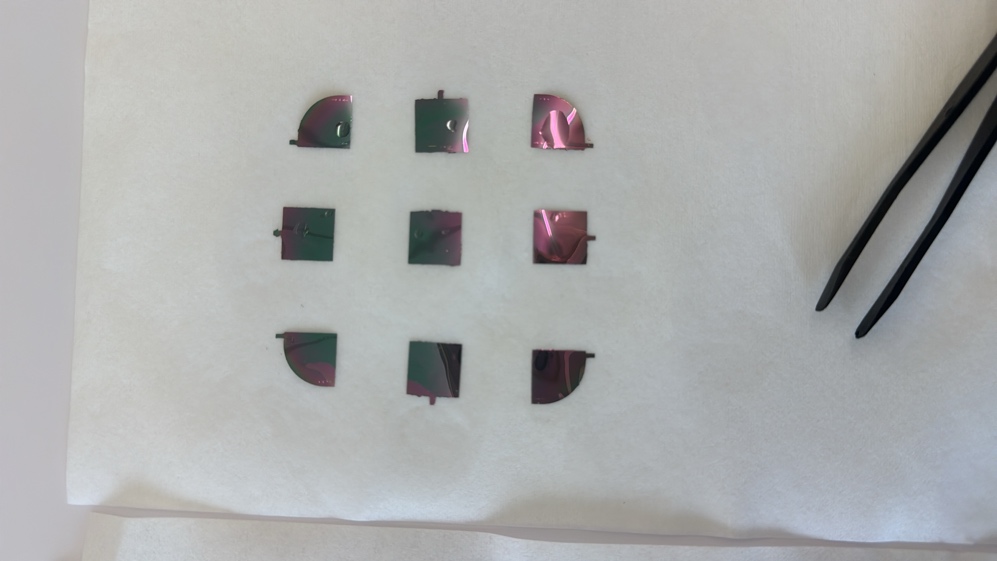

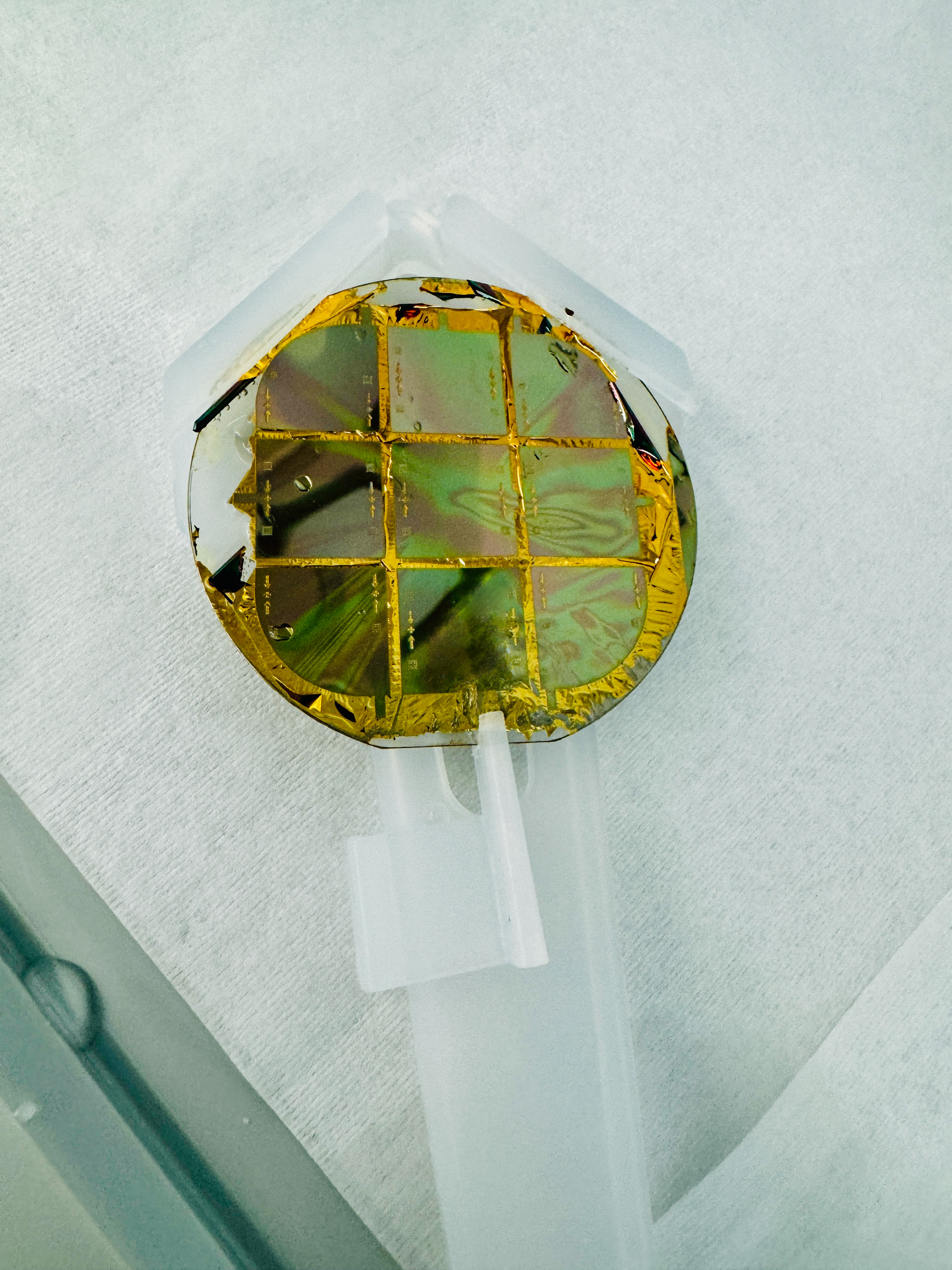


(a)

(c)

***Figure S1:*** *SLOT process main steps for the fabrication of nine 12X12 cm2 III-N MEMS resonator chips on flexible metal substrates by combining Van der Waals epitaxy of III-N epilayers on 2-inch sapphire growth substrate with the deposition of a thick metal stressor a top the III-N layers. (a) As grown III-N layer by Van der Waals Epitaxy on a Sapphire wafer. (b) After Ni metal stressor layer deposition. (c)* *Chip separation by stressor wet etching. (d) After Self Lift-off and Transfer.*

# **
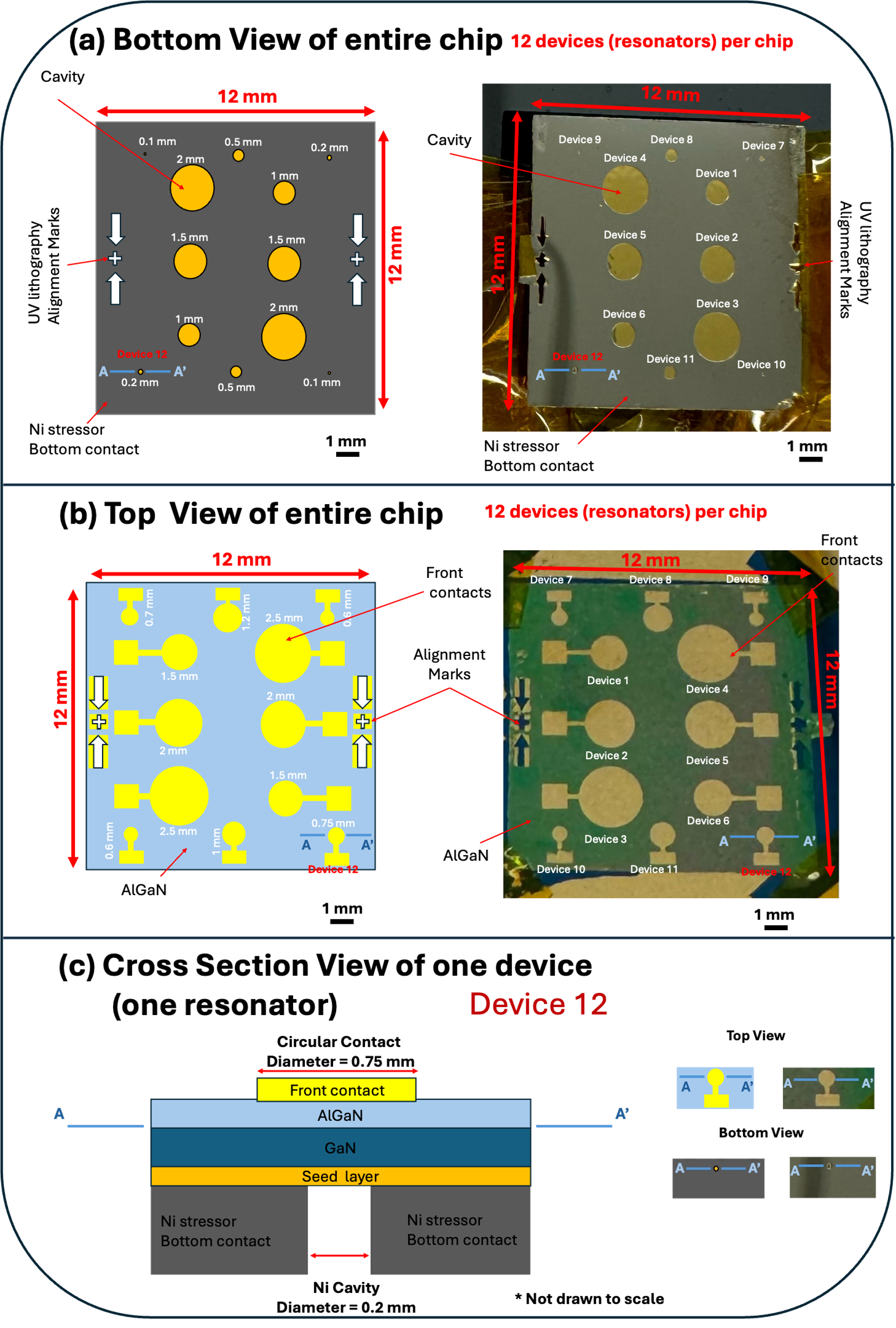
Section 2:** Design and structure of the fabricated resonators

**Figure S2:** Design schematics and corresponding optical micrographs of the fabricated chip containing 12 resonator devices. (a) Bottom view schematic (left) and optical micrograph (right) of the 12 mm × 12 mm chip layout, showing 12 circular Ni cavities with diameters ranging from 0.1 mm to 2 mm. (b) Top view schematic (left) and optical micrograph (right), highlighting the top-side metal structures, with the circular portions of the front contacts having diameters ranging from 0.6 mm to 2.5 mm, aligned with the underlying cavities. (c) Cross-sectional schematic along the A–A′ axis of Device 12, illustrating the internal layer structure, including the front contact, AlGaN/GaN stack, seed layer, and bottom Ni stressor/contact regions. Corresponding cross-sectional optical micrographs are shown to the right.

**
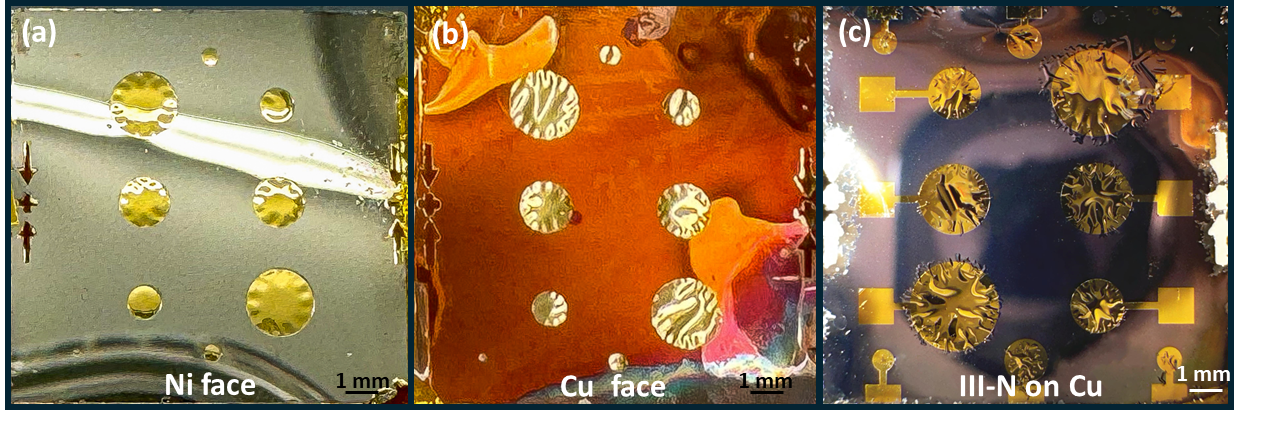
**

***Figure S3:*** *Released III-N membranes on a Nickel substrate observed from the Nickel side (a), and on a Copper substrate observed from Copper side (b) and from released exposed III-N layers side (c). The entire Copper chip tends to curl, resulting in observable differences in the orange hue of the Copper substrate in the photograph (b).*

**
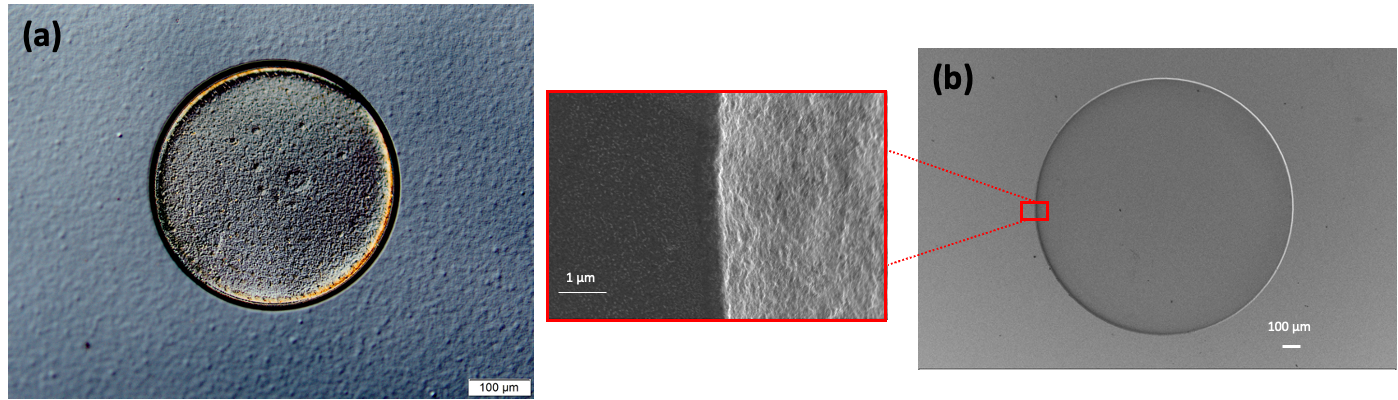
**

***Figure S4****: (a) Microscope optical images of a 500 µm diameter Nickel stressor cavity, showing the interface between the seed layer and the Nickel stressor. The orange color at the edges of the cavity indicates that the etching process is reaching the seed layer (Ti/Au).*

*(b) SEM image of a 1.5 mm Nickel cavity, with an inset showing the inclined sidewalls at the edge of the Nickel stressor cavity.*

# **Section 3:** Actuation mechanisms of the fabricated resonators
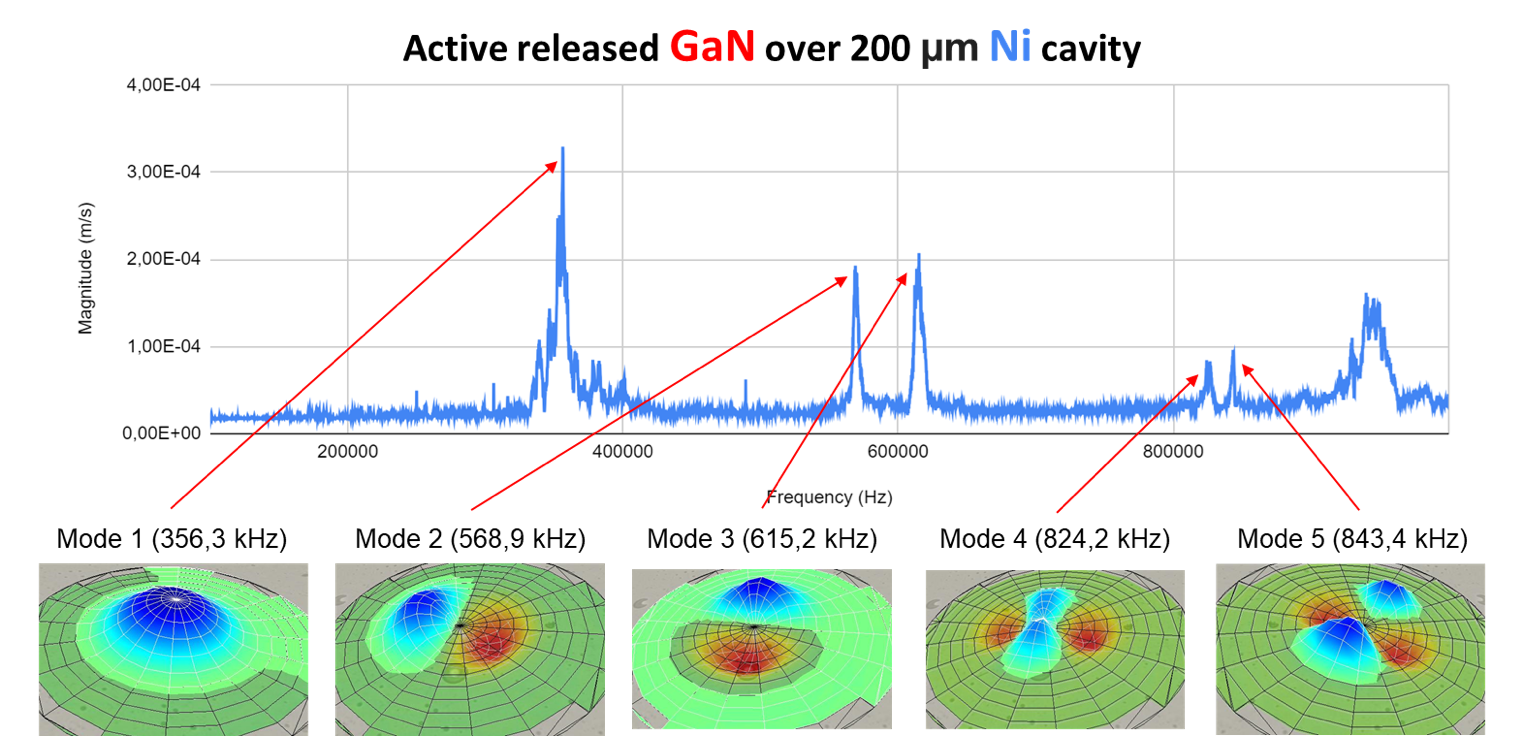


***Figure S5:*** *Typical resonance spectrum of a 340 nm thick and 209 µm in Nickel cavity diameter GaN resonator and its corresponding vibration modes. Amplitude of vibration is measured at the location of the largest displacement.*

The thermal power dissipation P is expressed as:

$\boldsymbol{P=}\boldsymbol{P}_{\boldsymbol{stat}}\boldsymbol{+}\boldsymbol{P}_{\boldsymbol{dyn 1}}\boldsymbol{+}\boldsymbol{P}_{\boldsymbol{dyn 2}}\boldsymbol{=}\frac{\boldsymbol{V}_{\boldsymbol{DC}}^{\boldsymbol{2}}}{\boldsymbol{R}}$ $\boldsymbol{+}\frac{\boldsymbol{V}_{\boldsymbol{AC}}^{\boldsymbol{2}}}{\boldsymbol{2}\boldsymbol{R}}$ $\boldsymbol{+}\frac{\boldsymbol{2}\boldsymbol{V}_{\boldsymbol{DC}}\boldsymbol{V}_{\boldsymbol{AC}}}{\boldsymbol{R}}\boldsymbol{cos(\omega t)}$ $\boldsymbol{+}\frac{\boldsymbol{V}_{\boldsymbol{AC}}^{\boldsymbol{2}}}{\boldsymbol{2}\boldsymbol{R}}\boldsymbol{cos(2}\boldsymbol{\omega t). (1)}$


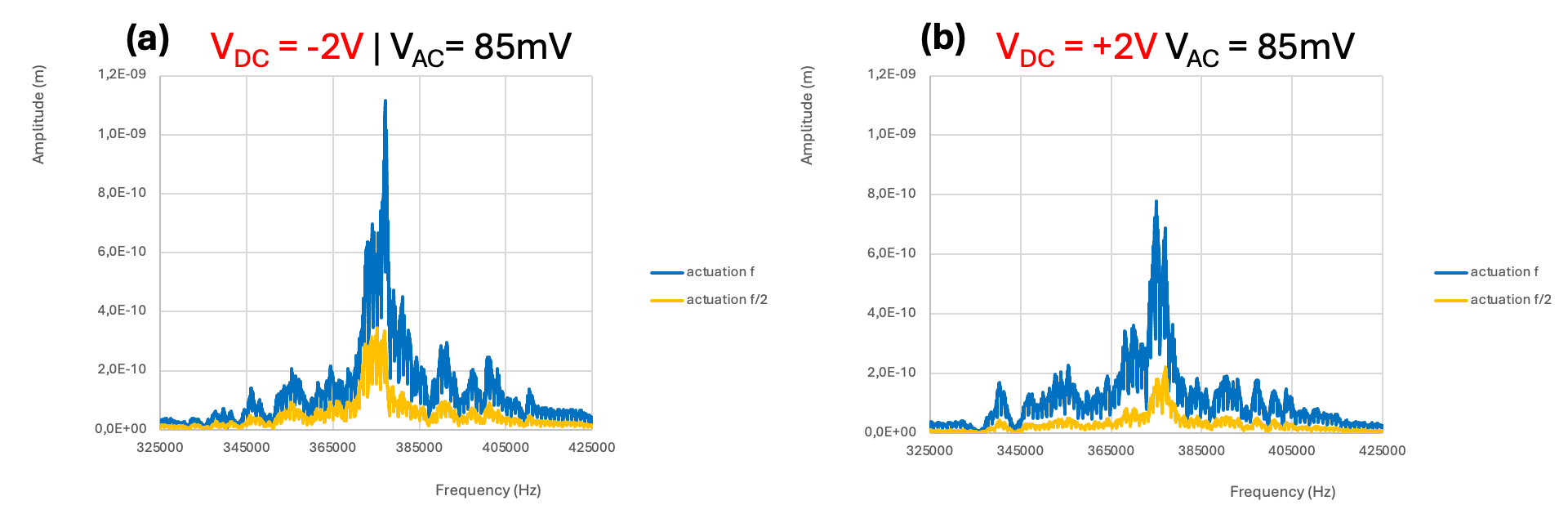
where V_DC_​ is the applied bias voltage, V_AC_​ is the excitation voltage, R is the resistance, and ω is the angular frequency of the AC signal.

**Figure S6:** Resonance frequency spectrum (maximum amplitude of vibration) of a 209 µm diameter GaN resonator excited at f and f/2 at (a) V_DC_ = -2V, V_AC_ = 85mV, and (b) V_DC_ = -2V, V_AC_ = 85mV.

According to this equation, if V_DC_ is nonzero then the electrothermal actuation results in resonances at both ω  and 2ω .

Figures S6(a) and S6(b) show frequency response spectra under different DC biases (V_DC_ = ±2V) with the same AC excitation (V_AC_ = 85mV).

The presence of a resonance peak at f when excitation is at f/2(orange curves in Fig. S6a and Fig. S6b) confirms an electrothermal actuation mechanism.

# **Section 4:** Quality factor and resonance of the fabricated resonators

**Figure S7:** Quality factors of the first five modes of a 107 μm diameter GaN resonator on Ni stressor.


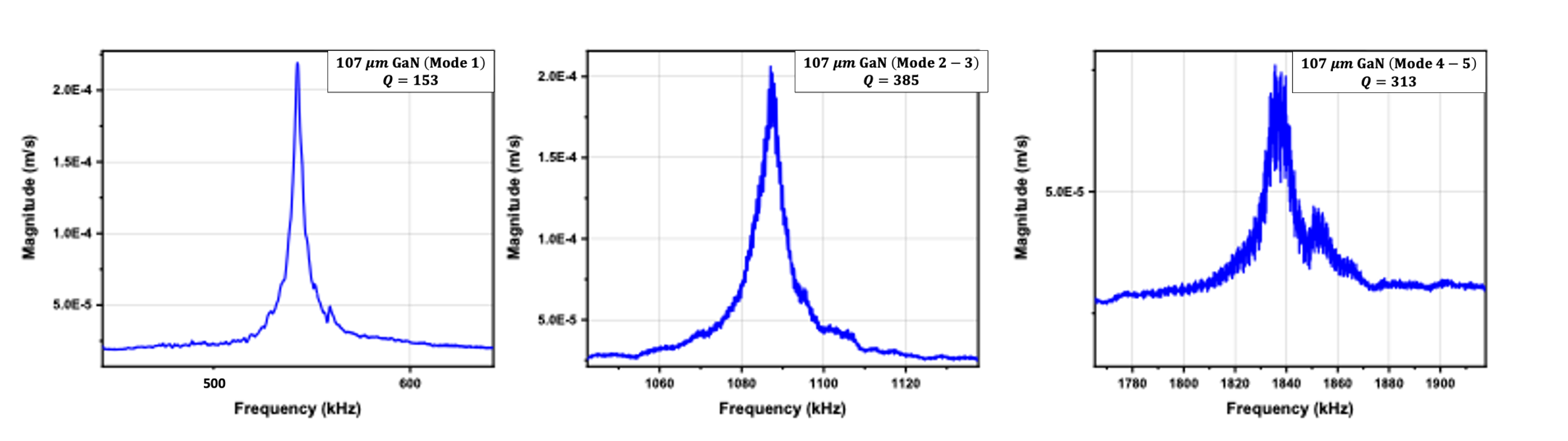


- For 107 $\boldsymbol{\mu m}$ diameter GaN resonator on Ni stressor, Q= 153 (Mode 1), Q= 385 (Mode 2-3), and Q= 313 (Mode 4-5).
- For 223 µm diameter AlGaN/ GaN resonator on Ni stressor, Q= 80 (Mode 1), Q =56 (Mode 2), Q=42 (Mode 3), Q= 178 (Mode 4), Q= 264 (Mode 5).

| **Chip** | **d average (µm)** | **Static deflection**  **(µm)** | **f1 (kHz)** | **f2 (kHz)** | **f3 (kHz)** | **f4 (kHz)** | **f5 (kHz)** |
| --- | --- | --- | --- | --- | --- | --- | --- |
| **GaN**  **on Ni** |  |  | **Experimental results by laser vibrometry** | | | | |
|  | 107 | 0 | 662±119 | 1208±121 | 1282±124 | 1836* | 1836* |
|  | 211.6±2.4 | 0.05±0.09 | 342±14 | 546±23 | 589±26 | 788±37 | 805±39 |
|  | 499.3±14.8 | 0.23±0.23 | 109±6 | 156±26 | 171±15 | 216±36 | 224±27 |
|  |  |  | **FEM simulation results with**  **s_GaN_ = 160 MPa (for d = 214 µm), s_GaN_ = 106 MPa ( for d = 500 µm)** | | | | |
|  | 214 |  | 321 | 529 | 529 | 737 | 737 |
|  | 514 |  | 103 | 166 | 166 | 227 | 227 |
|  | | | | | | | |
| **AlGaN/GaN**  **on Ni** |  |  | **Experimental results by laser vibrometry** | | | | |
|  | 124,5 | -1±0.31 | 214* | 630* | 630* | 1120* | 1120* |
|  | 223.3±0.3 | -1.4 | 238±30 | 372±42 | 441±27 | 653±16 | 673±4 |
|  | 550.8±26.8 | -4.6±0.12 | 89±13 | 114±1 | 114±1 | 139±1 | 139±1 |
|  |  |  | **FEM simulation results with**  **σ_GaN_ = 130 MPa and σ_AlGaN_ = -120 MPa** | | | | |
|  | 223 |  | 208 | 419 | 419 | 678 | 678 |
|  | 580 |  | 42 | 78 | 78 | 122 | 122 |

**Table S1:** Experimental and simulated values of resonant frequencies. All experimental values are averaged from the results of two different membranes of the same diameter, except those marked with

*, which were obtained from a single membrane (for the smallest membranes: f4 and f5 are measured on a 107 µm diameter GaN membrane, f1-f5 are measured on a 130 µm diameter AlGaN/GaN membrane).
